# Recent Advances in Collaborative Scheduling of Computing Tasks in an Edge Computing Paradigm

**DOI:** 10.3390/s21030779

**Published:** 2021-01-24

**Authors:** Shichao Chen, Qijie Li, Mengchu Zhou, Abdullah Abusorrah

**Affiliations:** 1 Faculty of Information Tecnology, Macau University of Science and Technology, Macau 999078, China; shichao.chen@ia.ac.cn; 2 The State Key Laboratory for Management and Control of Complex Systems, Institute of Automation, Chinese Academy of Sciences, Beijing 100190, China; 3 School of Mechanical and Electrical Engineering and Automation, Harbin Institute of Technology, Shenzhen 518000, China; liqijie1998@163.com; 4 Department of Electrical and Computer Engineering, New Jersey Institute of Technology, Newark, NJ 07102, USA; 5 Department of Electrical and Computer Engineering, Faculty of Engineering, and Center of Research Excellence in Renewable Energy and Power Systems, King Abdulaziz University, Jeddah 21481, Saudi Arabia; aabusorrah@kau.edu.sa

**Keywords:** collaborative scheduling, edge computing, internet of things, limited resources, optimization, task offloading

## Abstract

In edge computing, edge devices can offload their overloaded computing tasks to an edge server. This can give full play to an edge server’s advantages in computing and storage, and efficiently execute computing tasks. However, if they together offload all the overloaded computing tasks to an edge server, it can be overloaded, thereby resulting in the high processing delay of many computing tasks and unexpectedly high energy consumption. On the other hand, the resources in idle edge devices may be wasted and resource-rich cloud centers may be underutilized. Therefore, it is essential to explore a computing task collaborative scheduling mechanism with an edge server, a cloud center and edge devices according to task characteristics, optimization objectives and system status. It can help one realize efficient collaborative scheduling and precise execution of all computing tasks. This work analyzes and summarizes the edge computing scenarios in an edge computing paradigm. It then classifies the computing tasks in edge computing scenarios. Next, it formulates the optimization problem of computation offloading for an edge computing system. According to the problem formulation, the collaborative scheduling methods of computing tasks are then reviewed. Finally, future research issues for advanced collaborative scheduling in the context of edge computing are indicated.

## 1. Introduction

With the increasing deployment and application of Internet of Things (IoT), more and more intelligent devices, e.g., smart sensors and smart phones, can access a network, resulting in a considerable amount of network data. Despite that their computing power is very rapidly increasing, they are unable to achieve real-time and efficient execution due to their limited computing resources and ever-demanding applications. When it faces highly complex computing tasks and services, cloud computing [1,2] can process these tasks to achieve device–cloud collaboration. In a cloud computing paradigm, users can rely on extremely rich storage and computing resources of a cloud computing center to expand the computing and storage power of devices, and achieve the rapid processing of computing-intensive tasks. Yet there are some disadvantages in the device–cloud collaboration mode, such as incurring high transmission delay and pushing network bandwidth requirement to the limit.

In order to solve the problems of cloud computing for data processing, edge computing [3,4] is put forward to provide desired computing services [5] for users by using computing, network, storage and other resources on edge, that is near a physical entity or data source. Compared with cloud computing, some applications of users in edge computing can be processed on an edge server near intelligent devices, thus significantly reducing data transmission delay and network bandwidth load required in edge-cloud collaboration. Eliminating long-distance data transmissions encountered in device–cloud computing brings another advantage to edge computing, i.e., the latter can more effectively guarantee user data security. As a result, it has become an important development trend to use edge computing to accomplish various computing tasks for intelligent devices [6,7]. These devices are called edge devices in this paper.

The traditional scheduling strategies of edge computing tasks are to offload all computing-intensive tasks of edge devices to an edge server for processing [8,9,10]. However, it may result in the waste of computing and storage sources in edge devices and cloud computing centers. In addition, many devices may access an edge server at the same time period. As a result, the server may face too many computing tasks, thus resulting in a long queue of tasks. This increases the completion time of all queued tasks, even causing the processing delay of tasks in the edge server to exceed that at the edge devices. On the other hand, many edge devices may be idle, resulting in a waste of their computing resources; and resource-rich cloud centers may be underutilized. To solve the above problems, we can combine a cloud center, edge servers and edge devices together to efficiently handle the computing tasks of edge devices via task offloading. According to the computing tasks’ characteristics, optimization objectives and system status, we should utilize the computing and storage resources of a cloud center, edge servers and edge devices, and schedule computing tasks to them for processing on demand. It can effectively reduce the load of edge servers and improve the utilization of resources, and reduce the average completion time of computing tasks in a system.

This paper focuses on the important problem of collaborative scheduling of computing tasks in an edge computing paradigm under IoT. It is noted that edge computing systems can be viewed as a special class of distributed computing systems. Traditional task scheduling in distributed computing focuses on distributing and scheduling a large task into multiple similarly powerful computing nodes and do not have task off-loading issues in edging computing [9,10,11,12]. Edging computing arises to handle an IoT scenario where edge devices are resource-constrained and relatively independent. In Section 2, we analyze the edge computing scenarios, and clarify their composition, characteristics and application fields. In Section 3, we analyze the computing tasks, and classify them together with factors influencing their completion. We formulate the optimization problem of computation offloading with multiple objective functions for an edge computing system in Section 4. Based on the computing scenarios, computation tasks and formulated optimization model, we survey and summarize the collaborative scheduling methods of computing tasks in Section 5. This work is concluded in Section 6 by indicating the open issues for us to build a desired collaborative scheduling system for edge computing.

## 2. Computing Scenarios

In IoT, computing resources on edge are mainly composed of edge devices and edge servers. In order to take the advantages of cloud centers, we also consider them as part of the whole system in task scheduling. In general, a cloud center contains a large number of computing servers with high computing power. It is very important to reasonably use the computing, storage, bandwidth and other system resources to process computing tasks efficiently. In this section, different computing scenarios are analyzed and summarized according to the composition of computing resources.

On the edge, we have an edge server, edge devices and an edge scheduler. The server can provide computing, storage, bandwidth and other resources to support computing services for the edge computing tasks. Edge devices can execute computing tasks and may offload such tasks to the server and other available/idle edge devices. They have computing, storage, network and other resources, and can provide limited computing services for edge computing tasks. They have much fewer resources than edge servers do. An edge scheduler receives the computing tasks offloaded by edge devices, and provides scheduling services for the edge computing tasks according to the resources and status of all edge servers and edge devices under its supervision. It is a controller to realize the collaborative scheduling between edge servers and edge devices. But it does not have to be in an edge computing system.

**Figure 1:**
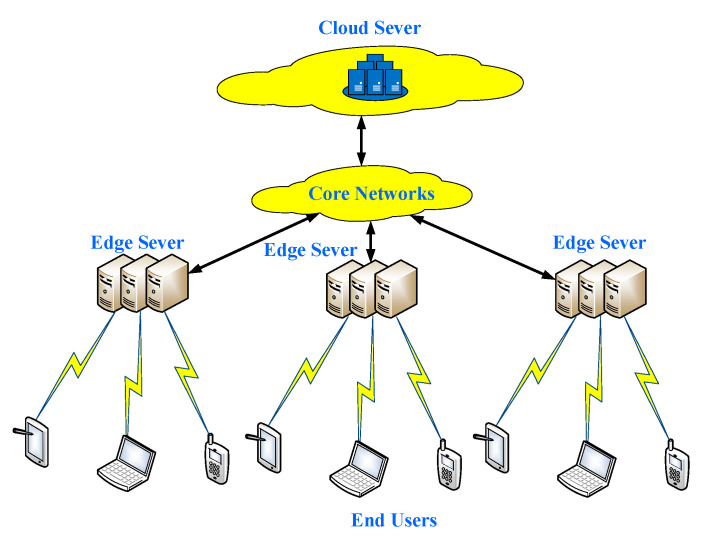
Edge computing architecture.

According to the difference among the computing resources involved in the offloading and scheduling of computing tasks in edge computing, computing scenarios can be divided into four categories, i.e., basic, scheduler-based, edge-cloud computing, and scheduler-based edge-cloud one. Their characteristics and application are described next. An edge computing architecture is shown in Figure 1.

### 2.1. Basic Edge Computing

**Figure 2:**
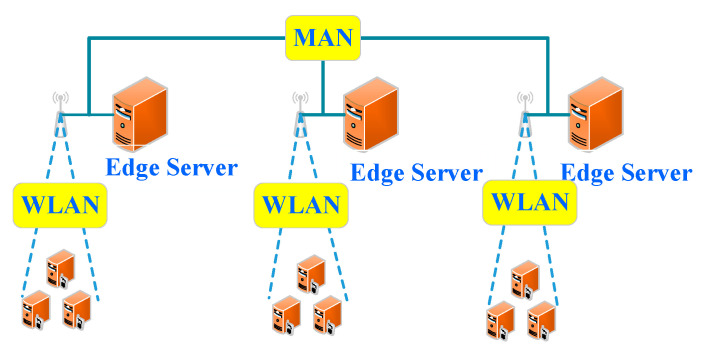
Basic edge computing (MAN: metropolitan area network and WLAN: wireless local area network).

The first scenario is composed of edge devices and edge servers. There is no edge scheduler on the edge. In this scenario, an edge device can execute a computing task locally, or offload it to its edge server. The edge server executes it, and then feeds back the computing result to the corresponding edge device. This scenario is similar to the scene that devices offload tasks to be performed in a cloud computing center. It is the simplest scenario in edge computing. There is no edge scheduler on the edge. For the computing tasks that can be offloaded to the edge servers, their offloading locations are fixed. Moreover, the processed types of computing tasks are fixed, and the specific types are determined by edge server resources. In addition, this scenario does not contain a cloud computing center. Hence, it is more suitable for processing tasks with a small amount of computation and strict delay requirements in a relatively closed environment. Its architecture is shown in Figure 2, which has been used in [11].

According to the above analysis, the Quality of Service (QoS) levels are expected to be achieved with the proposed scenario. Task completion time is used to measure QoS. We assume that all edge servers are same and all edge devices are uniform. Note that most of the presented content can be easily extended to heterogeneous devices and servers.

Let τ denote the completion time of a task offloaded to an edge server, which includes data transmission latency between an edge device and an edge server, and task processing time in an edge server and waiting time before it is processed. Let *η* denote the time to run a single instruction and *Ω* be the waiting time before a task to be processed in an edge server. G is the number of instructions for this task processing. Then the completion time of a task can be computed as:

(1) $$\tau =n\left(1+\;P_{\bar{\tau }\;}\right)\bar{\tau }\;+\Omega +\eta G\;$$

where n is the number of packets transmitted, which includes the process of bidirectional data transmission between an edge device and an edge server,$\;P_{\bar{\tau }\;}$ is the packet loss rate between an edge device and an edge server, occurring during n packet transmissions. $\bar{\tau }$ is the average latency per packet between an edge device and an edge server, which includes the sum of delays caused by processing, queuing, and transmission of n packets.

### 2.2. Scheduler-Based Edge Computing

**Figure 3:**
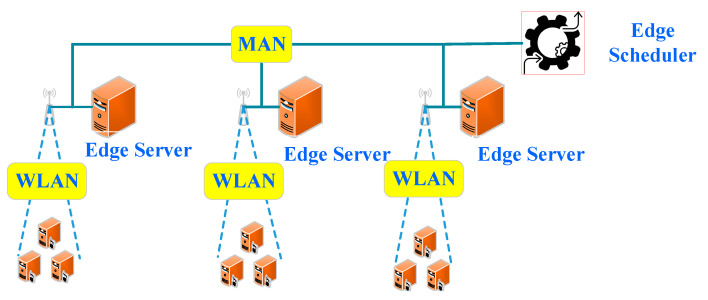
Scheduler-based edge computing.

The second one is composed of edge devices, edge servers and an edge scheduler. Compared with the first one, it includes an edge scheduler, which can schedule tasks strategically. In this scenario, an edge device can process a computing task locally or offload its task to an edge scheduler. The edge scheduler reasonably schedules the tasks to edge servers and edge devices according to scheduling policies. The policies are formed based on the current computing, storage, task execution status, network status, and other information related to all edge servers and devices. Finally, the scheduler feeds the computing results back to the source devices. The main feature of this scenario is that the computing tasks can be reasonably scheduled to different servers and edge devices by the edge scheduler, so that the collaborative processing of computing tasks in different edge servers and edge devices can be well-realized. The computing resources of edge devices can be fully utilized. The types of computing resources on the edge are diverse; so are the types of computing tasks that can be processed. The computing and storage resources on the edge are limited in comparison with a cloud computing center. Clearly, this architecture is suitable for processing tasks with a small amount of computation and strict delay requirements, as shown in Figure 3. The study [12] has adopted it.

According to this scenario, the task can be scheduled to an edge server for handling by an edge scheduler. Similar to the first scenario, the completion time of the task scheduled to a target edge server can be computed as:

(2) $$\rho =n\left(1+P_{\bar{\rho }}\right){\bar{\rho }}_{A}+n\left(1+P_{\bar{\rho }}{}^{\prime }\right){\bar{\rho }}_{B}+\Omega +\eta G\;$$

where $\;P_{\bar{\rho }}$ is the packet loss rate between an edge device and an edge scheduler, and $P_{\bar{\rho }}{}^{\prime }$ is the packet loss rate between an edge scheduler and a target edge server, occurring during n packets’ transmission. ${\bar{\rho }}_{A}$ is the average latency per packet between an edge device and an edge scheduler, and ${\bar{\rho }}_{B}$ is the average latency per packet between an edge scheduler and a target edge server, which includes the sum of delays caused by processing, queuing and transmission of n packets. If the task of an edge device is directly scheduled to its edge server, $P_{\bar{\rho }}$ = $P_{\bar{\tau }\;}$,$\;\bar{\tau }$ = ${\bar{\rho }}_{A}$, ${\bar{\rho }}_{B}=0$ and ρ =τ.

Let $\eta^{\prime }$ denote the time to run a single instruction and $G^{\prime }$ be the number of instructions for this task processing in an edge device. The completion time of the task scheduled to a target device can be computed as:

(3) $$\rho =n\left(1+P_{\bar{\rho }}\right){\bar{\rho }}_{A}+n\left(1+{P_{\bar{\rho }}}^{\prime \prime }\right){\bar{\rho }}_{C}+\Omega^{\prime }+\eta^{\prime }G^{\prime }$$

where $P_{\bar{\rho }}{}^{\prime \prime }$ is the packet loss rate between an edge scheduler and a target edge device, occurring during n packet transmissions. ${\bar{\rho }}_{C}$ is the average latency per packet between an edge scheduler and a target edge device, which includes the sum of delays caused by processing, queuing and transmission of n packets. $\Omega^{\prime }$ is the waiting time before a task is processed at an edge device.

Compared to the first scenario, the significant difference is that an edge scheduler can schedule the tasks to an idle edge server according to the network’s real-time status and edge servers. If the task processing time accounts for a large proportion of the total time, then it offers more advantages over the first one.

### 2.3. Edge-Cloud Computing

**Figure 4:**
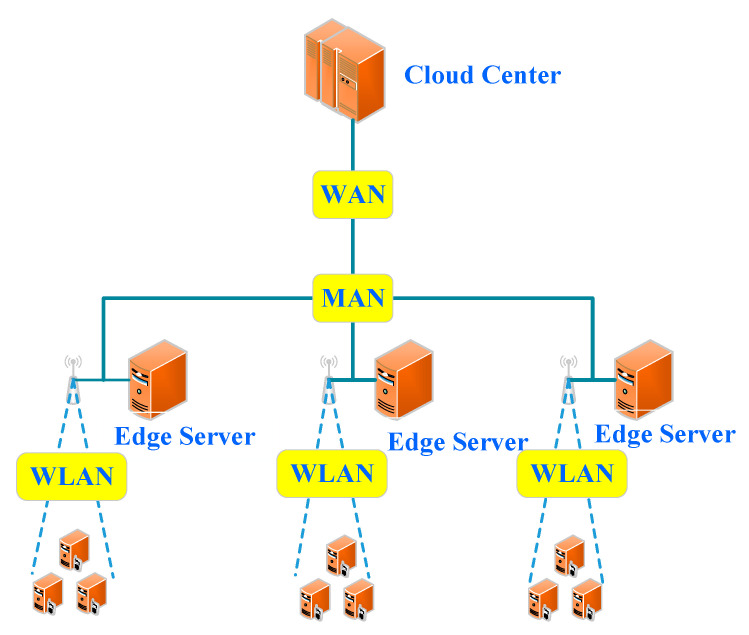
Edge-cloud computing.

This scenario is composed of edge devices, edge servers and a cloud computing center, and has no edge scheduler on the edge. An edge device can execute a computing task locally, or offload it to its edge sever or cloud center. Its difference from the first scenario is that its edge devices can offload their tasks to their cloud computing center. The specific offloading to an edge server or cloud computing center is determined by edge devices according to the attributes of their computing tasks and the QoS requirements from users. Its main feature is the same as the first scenario, i.e., the offloading location of an edge computing task and types are fixed. All the tasks that require a large amount of computation and are insensitive to delay on the edge can be offloaded to a cloud computing center. Therefore, the processing of computing tasks in this architecture is not affected by computing amount i.e., but only by the types of edge environment resources, as is shown in Figure 4. Such architectures are adopted in [13].

In this scenario, a task can be handled in an edge server or a cloud server. We formulize this scenario with the completion time, according to the location where the task is handled. The completion time of the task offloaded to an edge server is same as Equation (1).

Let ζ denote the completion time of a task offloaded to a cloud center, which includes data transmission latency between an edge device and a cloud center and task processing time in a cloud center. We assume that a cloud center is resource-intensive and the task computation does not need to wait. The completion time of a task can then be computed as follows:

(4) $$\zeta =n\left(1+P_{\bar{\zeta }}\right)\bar{\zeta }+\;\gamma G\;$$

where n is the number of packets transmitted, which includes the process of bidirectional data transmission between an edge device and a cloud center,$\;P_{\bar{\zeta }}$ is the packet loss rate between an edge device and a cloud center, occurring during n packets transmission, $\bar{\zeta }$ is the average latency per packet between an edge device and a cloud center, which includes the sum of delays caused by processing, queuing and transmission of n packets, and γ represents the time to run a single command in a cloud center.

### 2.4. Scheduler-Based Edge-Cloud Computing

**Figure 5:**
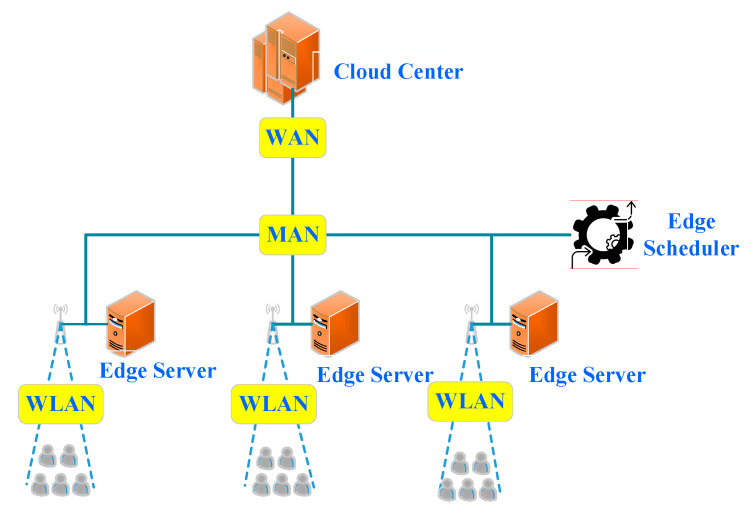
Scheduler-based edge-cloud computing.

The fourth scenario is composed of edge devices, edge servers, a cloud computing center and an edge scheduler. In this scenario, an edge device can execute its computing tasks locally or offload the tasks to the edge scheduler. Compared with the third architecture, the difference is that the edge scheduler receives all the computing tasks offloaded by edge devices, and schedules the computing tasks to proper computing entities (edge servers, idle edge devices, and/or a cloud computing center) for performing the services according to the computing resources, storage resources, network bandwidth and characteristics of tasks. It can give full play to the synergetic advantages among edge devices, edge servers and a cloud computing center. Its main feature is the same as the second one, i.e., the offloading position of edge computing tasks is uncertain, and the types are diverse. The processing of computing tasks is not affected by computing amount, but only by the types of edge environment resources. Its architecture is shown in Figure 5. It is used in [14].

In this scenario, the tasks can be handled in an idle edge server, a cloud server or an edge device. They can be scheduled to appropriate locations by an edge scheduler. Similar to the second scenario, the completion time of a task scheduled to a target edge server is the same as Equation (2). The completion time of a task scheduled to a target edge device is the same as Equation (3).

The completion time of a task scheduled to a cloud center can be computed as:

(5) $$\epsilon =n\left(1+P_{\bar{\epsilon }}\right){\bar{\epsilon }}_{A}+n\left(1+P_{\bar{\epsilon }}{}^{\prime }\right){\bar{\epsilon }}_{B}+\;\gamma G$$

where $P_{\bar{\epsilon }}$ is the packet loss rate between an edge device and an edge scheduler, and $P_{\bar{\epsilon }}{}^{\prime }$ is the packet loss rate between an edge scheduler and a cloud center, occurring during n packets transmission. ${\bar{\epsilon }}_{A}$ is the average latency per packet between an edge device and an edge scheduler and ${\bar{\epsilon }}_{B}$ is the average latency per packet between an edge scheduler and a cloud center, which includes the sum of delays caused by processing, queuing and transmission of n packets. 

Compared to other scenarios, the offloaded tasks can be scheduled to a suitable location by an edge scheduler according to the attributes of tasks and the real-time status of network, edge servers and cloud servers, which can give full play to the computing power of the whole network system to achieve the best QoS.

## 3. Computing Task Analysis

Next, the computing tasks are analyzed to ensure that they can be accurately scheduled to an appropriate node, and achieve the expected objectives, e.g., the minimal task completion time and least energy consumption. According to task attributes, we judge whether they can be split or not and whether there is interdependence among subtasks [15]. A specific judgment criterion is that if computing tasks are simple or highly integrated, they cannot be split, and they can only be executed locally as a whole at the edge devices or completely offloaded to edge servers. If they can be segmented based on their code and/or data [16,17], they can be divided into several parts, which can be offloaded. In summary, we have three modes, i.e., local execution, partial offloading and full offloading given computing tasks. The specific offloading location of computing tasks should be well considered according to the computing power of devices, current network status, and resource status of edge devices, edge servers and a cloud computing center.

### 3.1. Local Execution

Whether edge computing tasks are executed locally or not should be determined according to the resources of an edge device, edge servers’ network and resource status. If the available network bandwidth is not enough to support the successful uploading of a task, i.e., the remaining bandwidth of the current network is less than the bandwidth required for the uploading of the task, the computing task can only be performed locally. In addition, if the computing resources of edge servers are not available, resulting in that the computing tasks cannot be processed in time, the tasks have to be executed locally. If the computing power of an edge device itself can meet the service requirements, it performs its tasks locally, thus effectively reducing the workload of an edge server and the need for network bandwidth.

### 3.2. Full Offloading

Answering whether edge computing tasks are completely offloaded to an edge server or scheduler or not needs one to consider the resources of edge devices, current network, availability of edge servers’ resources and system optimization effect. If (1) the currently available network bandwidth supports the successful offloading of edge computing tasks, and (2) the edge servers or other edge devices are idle and the computing tasks that are successfully offloaded can be processed immediately, then, according to a scheduling goal, the results of local execution and full offloading to the edge servers are compared, and local execution or offloading of the computing tasks is decided. For example, if the goal is to minimize the completion time required for processing a task, it is necessary to compare the completion time required for local execution with the one required for offloading to an edge server/cloud computing center. If the local execution takes less time, the tasks should be processed locally. Otherwise, they should be offloaded to the edge servers or cloud computing center for processing.

### 3.3. Partial Offloading

An indivisible computing task at an edge device can only be executed locally or completely offloaded to the edge scheduler, which then assigns it to an appropriate edge server or idle edge device. Divisible computing tasks can enjoy partial offloading. Their split sub-tasks should be taken as a scheduling unit, and the resources of edge devices, network and the resources of edge servers should be considered comprehensively when they are scheduled. Considering the final processing effect of the overall tasks, each sub-task should be assigned to an appropriate computing node for processing. For the split computing task, if there are no interdependence among the sub-tasks, they can be assigned to different nodes to be processed at the same time so as to achieve the purpose of minimizing energy consumption and reducing task completion time. If there are some interdependence among the sub-tasks, the interdependent subtasks should be assigned to the same computing node for execution.

**Figure 6:**
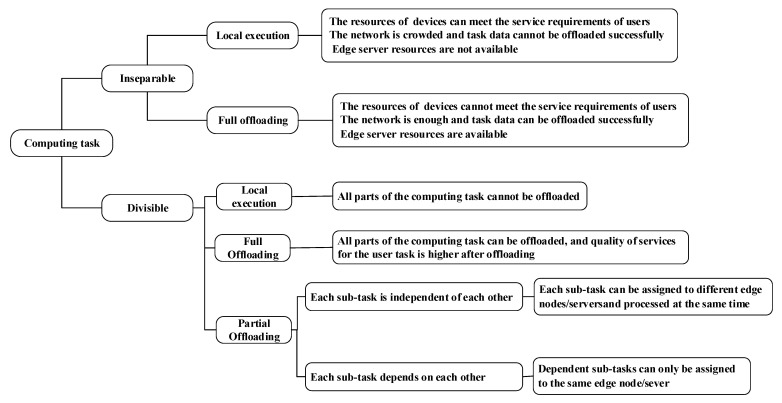
Computing task analysis and execution.

There are many methods for splitting tasks. Yang et al. [18] study the application repartition problem of periodically updating partition during application execution, and propose a framework for the repartition of an application in a dynamic mobile cloud environment. Based on their framework, they design an online solution for the dynamic network connection of cloud, which can significantly shorten the completion time of applications. Yang et al. [19] consider the computing partition of multi-users and the scheduling of offloading computing tasks on cloud resources. According to the number of resources allocated on the cloud, an offline heuristic algorithm called SearchAdjust is designed to solve the problem, thus minimizing the average completion time of a user’s applications. Liu et al. [20] make an in-depth study on the energy consumption, execution delay and cost of an offloading process in a mobile edge server system by using queuing theory, and put forward an effective solution to solve their formulated multi-objective optimization problems. The analysis result of computing tasks is summarized in Figure 6.

## 4. System Model and Problem Formulation

We can optimize multiple objective functions for computation offloading in an edge computing model. They include average or total delay time and energy consumption.

### 4.1. System Model

**Figure 7:**
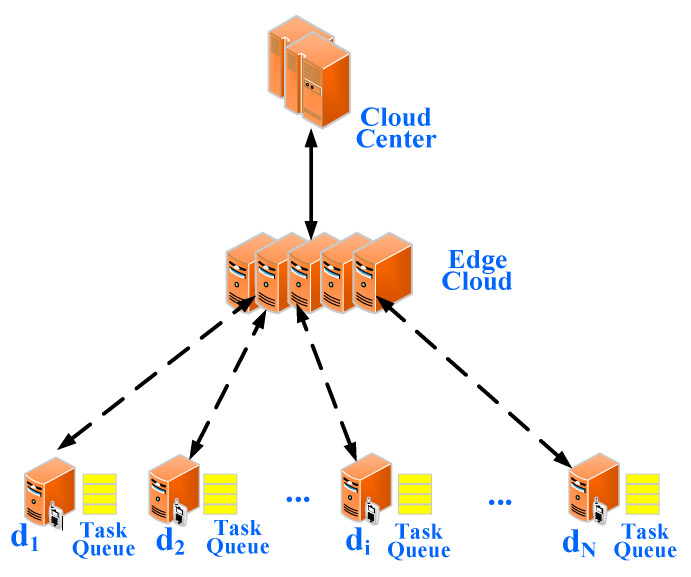
Edge computing model.

A typical model is shown in Figure 7. We assume that the system consists of N edge devices, an edge cloud and a cloud center. It is assumed that an edge cloud [21] consists of *n* edge servers powered on. We consider the queue model at an edge device as a M/M/1 queue, an edge cloud as M/M/n queue, and the cloud center as an M/M/∞ queue. Each edge device can offload a part or whole of its tasks to an edge cloud through a wireless channel. If the number of offloaded tasks exceeds the maximum one that an edge cloud can process, the edge cloud may further offload such overloaded tasks to the cloud center for processing.

In this computing system, let *D* = {$d_{1},d_{2},\ldots ,\;d_{N}$}, where *D* is the set of edge devices and $d_{i}$ is the ith edge device. let *S* = {$S_{1},S_{2},\ldots ,\;S_{n}$}, where $S_{k}$ is the kth edge server. We assume that the tasks generated by edge device $d_{i}$ obey a Poisson process with an average arrival rate $\;\lambda_{i}$ and contains data of size $\beta_{i}$. Let $u_{i}$ denote as an average service rate of edge device $d_{i}$. We denote $\vartheta_{i}$ as the probability of the edge device $d_{i}$ choosing to offload the task to an edge server. Then the tasks are offloaded to cloud follow a Poisson process with an average arrival rate $\vartheta_{i}\lambda_{i}$, and the tasks that are locally processed also follow a Poisson process with an arrival rate $\;\left(1-\vartheta_{i}\right)\lambda_{i}$. Let Q = {${\hat{Q}}_{1},{\hat{Q}}_{2},\ldots ,\;{\hat{Q}}_{N}$} where ${\hat{Q}}_{i}$ denotes the maximum task queue buffer of edge device $d_{i}$. We assume that an edge cloud has a single queue buffer and let ${\hat{Q}}_{C}$ denote its maximum task queue buffer. 

### 4.2. Communications Model

Let $h_{i}$ denote the channel power gain between edge device $d_{i}$ and an edge cloud. Denote $p_{i}$ as the transmission power of edge device $d_{i}$, 0 < $p_{i}$ <$\hat{p_{i}}$, where $\hat{p_{i}}$ is its maximum transmission power. The uplink data rate for computation offloading of edge device $d_{i}$ can be obtained as follows [22]:

(6) $$R_{i}=\kappa_{i}Blog_{2}\left(1+\frac{p_{i}h_{i}}{\sigma B}\right)$$

where B is the channel bandwidth, and σ denotes the noise power spectral density at the receiver, and $\kappa_{i}$ is the portion of bandwidth of an edge cloud’s channels allocated to edge device $d_{i}$, where $0\leq \kappa_{i}\leq 1$.

### 4.3. Task Offloading Model

(1) Local execution model

Let $w_{i}$ denote the normalized workload on the edge device $d_{i}$. It represents the percentage of central processing unit (CPU) that has been used. According a queue model M/M/1, we obtain the response time is T=1u1−ρ, where $\rho =\frac{\lambda }{u}$ is the utilization, λ is the task arrival rate of edge device $d_{i}$ [23]. We can compute the average response time of locally processing the tasks at edge device $d_{i}$ as follows:

(7) $$T_{i,o}=\frac{1}{u_{i}\left(1-w_{i}\right)-\left(1-\vartheta_{i}\right)\lambda_{i}}+\frac{1}{u_{i}}$$

where $0\leq w_{i}<1$ and $0\leq \left(1-\vartheta_{i}\right)\lambda_{i}\beta_{i}\leq {\hat{Q}}_{C}$.

The energy consumption of locally processing the tasks at edge device $d_{i}$ can be given as follows:

(8) $$E_{i,o}=P_{i}T_{i,\;o}$$

where $P_{i}$ denotes as computing power of edge device $d_{i}$ when the tasks are locally processed.

(2) Edge cloud execution model

According to above analysis, we can obtain the transmission time of offloading the tasks from edge device $d_{i}$ to an edge cloud as follows:

(9) $${\tilde{T}}_{i\;}=\frac{\vartheta_{i}\lambda_{i}\beta_{i}}{R_{i}}$$

In (9), $0\leq \vartheta_{i}\lambda_{i}\beta_{i}\leq {\hat{Q}}_{C}\;$.

Let ${\tilde{E}}_{i}$ denote the energy consumption of transmitting the tasks from edge device $d_{i}$ to an edge cloud, and it is as follows:

(10) $${\tilde{E}}_{i}=p_{i}\;{\tilde{T}}_{i\;}$$

According to the queue model M/M/n, let χ = {$\chi_{1},\chi_{2},\ldots ,\;\chi_{n}$}, where $\chi_{k}$ is the service rate of the kth edge server. We use $f_{k}$ to denote the CPU cycle frequency and ${\hat{\omega }}_{k}$ to denote the maximum workload capacity of the kth edge server. Let $\hat{\lambda }$ denote the maximum task accepted rate of the edge cloud.

The total task arrival rate from  N edge devices to an edge cloud can be computed as:

(11) $$\lambda_{all}=\overset{N}{\underset{i=1}{\sum }}\vartheta_{i}\lambda_{i}$$

Then the fraction of tasks φ that the edge cloud can compute is be given:

(12) $$\varphi =\{\begin{array}{c} 1\;\hat{\lambda }\geq \;\lambda_{all}\;\\ \frac{\;\hat{\lambda }}{\lambda_{all}}\;\hat{\lambda }<\lambda_{all}\; \end{array}$$

Hence, the actual execution rate at the edge cloud can be denoted as:

(13) $$\tilde{\lambda }=\varphi \lambda_{all}$$

We can get the average waiting time of each task at an edge cloud.

(14) $$T_{i,\;W}=\frac{S}{\sum_{k=1}^{n}\chi_{k}-\tilde{\lambda }}$$

where S  denotes the utilization of an edge cloud.

The average computing time of each task is:

(15) $$T_{i,C\;}=\frac{n}{\sum_{k=1}^{n}\chi_{k}}$$

According to [24], the workload of each edge server cannot exceed its maximum workload, and we can obtain the constraints:

(16) $$0<\frac{f_{k}}{\chi_{k}}\leq {\hat{\omega }}_{k}$$

Let $u_{r}$ denote the transmission service rate of an edge cloud, we can get the expected waiting time for the computation results:

(17) $${\bar{T}}_{i}=\frac{1}{u_{r}-\tilde{\lambda }}$$

Similar to many studies [25,26], because the amount of data for the computation results is generally small, we ignore the transmission delay and energy consumption for an edge cloud to send the results back to an edge device. 

We can get the energy consumption of edge device $d_{i}$ when the offloaded tasks of edge device $d_{i}$ are processed:

(18) $$E_{i,\;W}=p_{i}^{\prime }(T_{i,W}+T_{i,C\;}+{\bar{T}}_{i})$$

where $p_{i}^{\prime }$ is the computation power of edge device $d_{i}$ after offloading tasks.

Let ${\overset{=}{T}}_{i}$ denote the total time of an offloading process from the task transmission of edge device $d_{i}$ to the computation results returned of an edge cloud. We have:

(19) $${\overset{=}{T}}_{i}={\tilde{T}}_{i\;}+T_{i,\;W}+T_{i,C\;}+{\bar{T}}_{i}$$

The energy consumption of offloading process at edge device $d_{i}$ can be obtained as:

(20) $${\overset{=}{E}}_{i}={\tilde{E}}_{i}+E_{i,\;W}$$

(3) Cloud execution model

When $\lambda_{all}>\hat{\lambda }$ is realizable, the overloaded tasks are transmitted to the cloud center by wire network. Let $T_{N}$ denote a fixed communication time between an edge server and a data center. Due to the enough computing power of the cloud center, we assume no task waiting time at the cloud center. According to the queue model M/M/∞, let $u_{CC}\;$ denote the service rate of a cloud center. The execution time of an overloaded task is:

(21) $$T_{i,CC\;}=T_{N}+\frac{1}{u_{CC}}$$

The expected time for the results of overloaded tasks from a cloud center back to the corresponding edge device:

(22) $$T_{i,CC}^{\prime }=\frac{1}{u_{r}-\tilde{\lambda }}+T_{N\;}$$

Let ${\overset{=}{T}}_{i,CC}$ denote the total time of an offloading process from the task transmission of edge device $d_{i}$ to the computation results returned from a cloud center. We have:

(23) $${\overset{=}{T}}_{i,CC}=T_{i,CC\;}+T_{i,CC}^{\prime }$$

The corresponding energy consumption is:

(24) $${\overset{=}{E}}_{i,CC}=p_{i}^{\prime }\;(T_{i,cc\;}+T_{i,CC}^{\prime })$$

### 4.4. Problem Formulation

From (7), (8), (14), (15), (17), (21) and (22), we can obtain the execution delay time for edge device $d_{i}$, i.e.,

(25) $$T_{i}=T_{i,\;o}+{\tilde{T}}_{i\;}+\varphi \left(T_{i,W}+T_{i,C\;}+{\bar{T}}_{i}\right)+\left(1-\varphi \right)(T_{i,CC\;}+T_{i,CC}^{\prime })$$

So, the average delay time of all edge devices in the computing system is:

(26) $$\bar{T}=\frac{1}{N}\overset{N}{\underset{i=1}{\sum }}T_{i}$$

To minimize the total execution time of these tasks in edge computing system, we formulate the optimization problem as:

(27) $$Min\{\bar{T}\}$$

subject to:

(28) $$(1-\vartheta_{i})\lambda_{i}<u_{i}\left(1-w_{i}\right)$$

(29) $$0\leq \left(1-\vartheta_{i}\right)\lambda_{i}\beta_{i}\leq {\hat{Q}}_{i}$$

(30) $$0\leq \vartheta_{i}\lambda_{i}\beta_{i}\leq {\hat{Q}}_{C}$$

(31) $$\tilde{\lambda }<\overset{n}{\underset{k=1}{\sum }}\chi_{k}$$

(32) $$0<p_{i}\;>\hat{p_{i}}$$

(33) $$0\leq \vartheta_{i}\leq 1$$

(34) $$0\leq w_{i}<1$$

(35) $$0\leq \theta_{i}\leq 1$$

and (16).

From (8), (10), (18) and (24), we can get the energy consumption for edge device $d_{i}$, which is given as follows:

(36) $$E_{i}=E_{i,\;o}+{\tilde{E}}_{i}+\varphi E_{i,\;W}+\left(1-\varphi \right){\overset{=}{E}}_{i,CC}$$

So, the average energy consumption of all edge devices in the computing system are denoted as follows:

(37) $$\bar{E}=\frac{1}{N}\overset{N}{\underset{i=1}{\sum }}E_{i}$$

To minimize the energy consumption of these tasks in an edge computing system, we formulate the optimization problem as:

(38) $$Min\left\{\bar{E}\right\}$$

subject to: (16) and (28)–(35).

Min{$\bar{T}$} and $Min\left\{\bar{E}\right\}$ together with the related constraints lead to a biobjective optimization problems. It can be solved with different methods [27,28,29].

Problem (27) represents a constrained mixed integer non-linear program to make the optimal offloading decisions. Its exact optimal solution complexity is NP-hard, and cannot be solved with polynomial-time methods. We can obtain an optimal solution in some specific scenarios with polynomial-time ones, but not in general cases as shown in [22,24,30].

## 5. Computing Task Scheduling Scheme

After thoroughly analyzing computing tasks in edge computing, the tasks offloaded to an edge scheduler need to be synergistically scheduled. Due to the limited computing and storage resources on the edge devices, and the resource competition among multiple tasks, it is essential in scheduling the tasks optimally in terms of task completion time and energy consumption [31].

Many scheduling algorithms have been proposed. Traditional task scheduling algorithms mainly include Min-Min, Max-Min, and Sufferage algorithm [32], first come first served, and minimum completion time [33]. Most of them take delay as an optimization goal, but it is easy to result in the problem of load imbalance among computing nodes. Intelligent heuristic task scheduling algorithms mainly include Genetic Algorithm (GA), Ant Colony Optimization, Particle Swarm Optimization (PSO), Simulated Annealing (SA), Bat algorithm, artificial immune algorithm, and Tabu Search (TS) [34,35]. These algorithms are based on heuristic rules to quickly get the solution of a problem, but they cannot guarantee the optimality of their solutions [36]. In the scheduling processes of edge computing tasks, we face many goals. We summarize the methods of task scheduling with aims to achieve the lowest delay and/or lowest energy consumption.

### 5.1. Minimal Delay Time

The completion time of computing tasks offloaded to edge servers is mainly composed of three parts, the transmission time required to transmit the tasks to the edge servers or edge devices, the processing time required to execute the tasks in the edge server or edge devices and the time required to return the result after the completion of the task processing. Therefore, reducing the above three parts of task completion time can effectively improve QoS.

Yuchong et al. [24] propose a greedy algorithm to assign tasks to servers with the shortest response time to minimize the total response time of all tasks. Its experimental results show that the average response time of tasks is reduced in comparison with a random assignment algorithm. In intelligent manufacturing, a four-layer computing system supporting the operation of artificial intelligence tasks from the perspective of a network is proposed in [37]. On this basis, a two-stage algorithm based on a greedy strategy and threshold strategy is proposed to schedule computing tasks on the edge, so as to meet the real-time requirements of intelligent manufacturing. Compared with the traditional algorithm, the experimental results show that it has good real-time performance and acceptable energy consumption. A Markov decision process (MDP) method is used to schedule computing tasks according to the queuing state of task buffers, the execution state of a local processing unit and the state of the transmission unit. Its goal is to minimize delay while enforcing a power constraint. Its simulation results show that compared with other benchmark strategies, the proposed optimal random task scheduling strategy has shorter average execution delay [22]. Zhang et al. [38] study a task scheduling problem based on delay minimization, and establish an accurate delay model. A delay Lyapunov function is defined, and a new task scheduling algorithm is proposed. Compared with the traditional algorithm depending on Little’s law, it can reduce the maximum delay by 55%. Zhang et al. [39] model the delay of communication and computing queue as a virtual delay queue. A new delay-based Lyapunov function is defined, and joint subcarrier allocation, base station selection, power control and virtual machine scheduling algorithms are proposed to minimize the delay. Zhang et al. [40] propose an optimization model of maximum allowable delay considering both average delay and delay jitter. An effective conservative heterogeneous earliest completion time algorithm is designed to solve it. Yuan et al. [41] jointly consider CPU, memory, and bandwidth resources, load balance of all heterogeneous nodes in the edge layer, the maximum amount of energy, the maximum number of servers, and task queue stability in the cloud data centers layer. It designs a profit-maximized collaborative computation offloading and resource allocation algorithm to maximize the profit of the system while ensuring that response time limits of tasks are strictly met. Its simulation results show better performance than other algorithms, i.e., firefly algorithm and genetic learning particle swarm optimization.

In the present research, treating the minimum delay as a scheduling goal, most researchers have improved some traditional task scheduling algorithms by using intelligent optimization algorithms. The reason why these algorithms are not chosen in practical use is that they need multiple iterations to derive a relatively high-quality solution. However, facing many tasks or online application scenarios of random tasks, their execution may introduce delay. 

### 5.2. Minimal Energy Consumption

The energy consumption of computing tasks is mainly composed of two parts, including energy for their processing, transmission from edge devices to an edge server, and returning results to the source node [36]. Therefore, on the premise of meeting the delay requirements of tasks, energy consumption should be minimized.

Xu et al. [30] propose a particle swarm optimization algorithm for the scheduling of tasks that can be offloaded to edge servers. It considers different kinds of computing resources in a Mobile Edge Computing (MEC) environment and aims to reduce mobile devices’ energy consumption under response time constraints. Their experimental results show that it has stable convergence and optimal adaptability, and can effectively achieve the optimization goal. A heuristic algorithm based on MEC for efficient energy scheduling is proposed in [42]. The task scheduling among MEC servers and the downlink energy consumption of roadside units are comprehensively considered. The energy consumption of MEC servers is minimized while enforcing task delay constraints. The algorithm can effectively reduce the energy consumption, task processing delay and solve the problem of task blocking. Li et al. [43] study the energy efficiency of an IoT system under an edge computing paradigm, and describes a dynamic process with a generalized queuing network model. It applies the ordered optimization technology to a Markov decision-making process to develop resource management and task scheduling schemes, to meet the challenge of the explosion in Markov decision process search space. Its simulation results show that this method can effectively reduce the energy consumption in an IoT system. A dynamic voltage frequency scale (DVFS) technology is proposed in [44], which can adjust the offloading rate of a device and the working frequency of CPU to minimize the energy consumption under the constraint of time delay. Zhang et al. [45] propose a dual depth Q-learning model. A learning algorithm based on experience playback is used to train model parameters. It can improve training efficiency and reduces system energy consumption. An improved probability scheme is adopted to control the congestion of different priority packets transmitted to MEC in [46]. Based on this, an improved krill herd meta-heuristic optimization algorithm is proposed to minimize the energy consumption and queuing congestion of MEC. Bi et al. [47] propose a partial computation offloading method to minimize the total energy consumed by smart mobile devices (SMDs) and edge servers by jointly optimizing offloading ratio of tasks, CPU speeds of SMDs, allocated bandwidth of available channels and transmission power of each SMD in each time slot. They formulate a nonlinear constrained optimization problem and presents a novel hybrid meta-heuristic algorithm named genetic simulated-annealing-based particle swarm optimization (GSP) to find a close-to-optimal solution for the problem. Its experimental results prove that it achieves lower energy consumption in less convergence time than other optimization algorithms including SA-based PSO, GA and SA.

Based on the current research, treating the lowest energy consumption as the scheduling goal, researchers have proposed many improved heuristic task scheduling algorithms. For cases where a delay constraint is not strong, most of the heuristic scheduling algorithms are able to generate a complete feasible schedule by gradually expanding a local schedule. The more iterations, the greater chance to get the best solution, and the lower energy consumption.

### 5.3. Minimal Delay Time and Energy Consumption

In such application scenarios as virtual reality, augmented reality, and driverless vehicles, the requirements of delay time and energy consumption are very strict. How to make an optimal task schedule to minimize both time delay and the energy consumption is very important. Two objectives are, unfortunately, in conflict with each other.

The energy consumption and processing/transmission time of computing tasks are regarded as costs. With the support of a cloud computing center, a distributed algorithm for cost minimization is proposed by optimizing the offloading decision and resource allocation of a mobile edge computing system [48]. Its experimental results show that compared with other existing algorithms, i.e., Greedy algorithm, and those in [49,50], the cost can be reduced by about 30%. Zhang et al. [51] study the trade-off between system energy consumption and delay time. Based on the Lyapunov optimization method, the optimal scheduling of CPU cycle frequency and data transmission power of mobile devices is performed, and an online dynamic task allocation scheduling method is proposed to modify the data backlog of a queue. A large number of simulation experiments show that the scheme can realize good trade-off between energy consumption and delay. A task scheduling problem of a computing system considering both time delay and energy consumption is proposed in [52]. A task allocation method based on reinforcement learning is proposed to solve the problem, which can ensure the timely execution of tasks and a good deal of efficient energy saving. The simulation results show that compared with other existing methods, i.e., SpanEdge [53] and suspension-and energy-aware offloading algorithm [54], it can reduce 13–22% of task processing time and 1–10% of task processing energy consumption. Note that Sen at al [52] fail to consider the transmission energy in such a system.

In the existing research, a distributed algorithm, Lyapunov optimization method, reinforcement learning and other task scheduling algorithms can be used to improve the overall performance of the system with a target to lower both delay time and energy consumption. It can be seen that to balance both well, engineers can select traditional and heuristic task scheduling algorithms. The latter are more popular since they can handle dual objective functions well. 

All the discussed scheduling schemes and other methods [55,56,57,58,59,60,61] are summarized in Table 1 and Table 2.

## 6. Issues and Future Directions

An edge server has more computing power and storage capacity than devices, and edge computing has lower task transmission delay than cloud computing. In addition, due to the limitation of edge resources, the task scheduling problem of edge computing is NP-hard. Its high-performance solution methods are highly valuable, while its exact global optimal solution cannot be obtained in general for sizable problems. Although there have been many studies on collaborative scheduling of computing tasks in edge computing [47,62,63], the following issues should be addressed: 

(1) Consider the occurrence of emergencies. In an edge computing paradigm, a system involves the coordination of devices, edge server and network link, each of which plays an important role. Therefore, if any devices and edge servers are shut down or the network fails in the process of task processing, scheduled task execution can be significantly impacted. Therefore, the question of how to add the consideration of emergencies in the process of task scheduling to ensure that tasks can also be successfully executed is a widely open problem.

In other words, researchers have to take their failure probability into tasks scheduling consideration so that the risk of failing some important tasks should be minimized.

(2) Consider multiple optimization objectives. Now, most of research is based on the optimization goals of delay time and/or energy consumption to develop task schedules. Other QoS indicators of user tasks are rarely considered. Therefore, they should be added to optimize a schedule, and a task scheduling scheme with comprehensive optimization goals should be formulated to achieve high-quality user service experience as well.

(3) Consider data security issues. Data security [64,65,66,67,68,69] is one of the most concerned issues. Security protocols and encryption algorithms are mostly used to achieve the security and privacy of data, but there are few considerations in terms of their induced delay and energy consumption issues. Therefore, it is worthy to develop light to heavy-weight security protocols and encryption algorithms such that some best trade-off solutions between performance and data security levels can be made.

(4) Find any-time task scheduling algorithms. The research on task scheduling algorithms mostly uses the improved traditional and heuristic task ones. They need long iteration time to achieve a near-optimal or optimal schedule. In practice, we must offer a feasible schedule in short time. We can then improve it if we are given enough computing time before a schedule needs to be developed. Hence, inventing some fast algorithms to produce a first feasible schedule and then some intelligent optimizations that can improve the solutions are highly desired.

(5) Add some important factors to optimization goals. Generally, network bandwidth and CPU of task offloading locations could be taken into consideration in the process of offloading tasks at the edge, but many other factors, such as offloading ratio of tasks, are not yet taken into consideration in order to obtain the best offloading strategy.

(6) Balance partial computing offloading of Deep Learning (DL) models. To improve the intelligence of the applications, DL is increasingly adopted in various areas, namely face recognition, natural language processing, interactive gaming, and augmented reality.

Due to the limited resources of edge hardware, lightweight DL models are suitable for edge devices. However, in order to accelerate the inference speed of models and minimize the energy consumed by devices, they need to be developed and partially offloaded. It is challenging to answer how to determine partial offloading for DL model training and balance the resource consumption between edge devices and edge servers.

## 7. Conclusions

This paper analyzes and summarizes the computing scenarios, computing tasks, optimization objectives’ formulation and computing task scheduling methods for the scheduling process of an edge computing system. According to the resources in edge computing, the computing scenarios of scheduling tasks are divided into four categories, and their composition and characteristics are analyzed in detail. According to where their execution takes place, computing tasks can be accomplished via local execution, partial offloading and full offloading. Then we formulate the optimization problem to minimize delay time and energy consumption for computation offloading of an edge computing system with different queuing models, and indicate its solution complexity. With regard to computing task scheduling methods in edge computing, most existing studies set their optimization goal to minimize delay, energy consumption or both of them. Improved traditional task scheduling algorithms and some intelligent optimization algorithms can be used to solve such optimization problem. For the reviewed optimization problems, most researchers tend to use the improved heuristic algorithm/intelligent optimization algorithms instead of mathematical programing ones due to their computational complexity. This paper also discusses the issues and future directions in the area of collaborative scheduling of computing tasks in an edge computing paradigm. This paper should stimulate further research on collaborative scheduling and its applications in the context of edge-computing, e.g., [70,71,72].

## Figures and Tables

**Table 1:** Summary of task scheduling schemes.

Objectives	Studies	Features
Minimize delay time	[22,24,37,38,39,40,41]	Markov decision process method Greedy Algorithm Two-stage algorithm based on greedy strategy and threshold strategy Establishing the delay model of cellular edge computing system and defining the delay Lyapunov function Defining D Lyapunov function and proposing the algorithm of joint subcarrier allocation, base station selection, power control and virtual machine scheduling Conservative heterogeneous earliest completion time algorithm A profit-maximized collaborative computation offloading and resource allocation algorithm to guarantee the response time limits of tasks.
Minimize energy consumption	[30,42,43,44,45,46,47]	Particle swarm optimization-based task scheduling algorithm for multi-resource computing offloading Heuristic algorithm of task scheduling among Mobile Edge Computing (MEC) servers by considering the downlink energy consumption of Road Side Units Applying ordered optimization technology to a Markov decision process Based on Dynamic Voltage Frequency Scale (DVFS) Technology Double Deep Q-learning model Improved krill herd meta heuristic optimization algorithm A novel genetic simulated-annealing-based particle swarm optimization (GSP) algorithm to produce a close-to-optimal solution
Minimize both delay time and energy consumption	[48,51,52]	Dynamic task allocation and scheduling algorithm based on a Lyapunov optimization method Distributed algorithm Reinforcement learning

**Table 2:** Summary of algorithms for different optimization objectives (ES = edge server, CC = cloud center, Unc = uncertain and N-O = near-optimal).

Objective	Scheme	Optimal	Complexity	Where	Pros and Cons
Delay Time	One-dimensional search algorithm [22]	Yes	low	ES	Achieving the minimum average delay in various specific scenarios, but not general ones
	Greedy algorithm [24]	Yes	Medium	ES	Saving time by 20–30%, in comparison to the proposed random algorithm but only for a simple M/M/1 queuing system in a specific scenario.
	Customized TS algorithm [24]	Yes	Medium	ES	Efficient and suitable for scenarios with large number of tasks, but only for a simple M/M/1 queuing system in a specific scenario.
	Lyapunov function-based task scheduling algorithm [38]	NP	Medium	ES	Being more accurate than the other delay models; and smaller delay than that of a traditional scheduling algorithm.
	Efficient conservative heterogeneous earliest-finish-time algorithm [40]	Unc	Medium	ES	Reducing the delays of task offloading, and considering the task execution order.
	SA-based migrating birds optimization procedure [41]	N-O	Medium	ES/CC	Providing a high-accuracy and fine-grained energy model by jointly considering central processing unit (CPU), memory, and bandwidth resource limits, load balance requirements of all nodes, but only for a simple M/M/1 system.
	Sub-gradient algorithm [55]	Yes	Medium	ES	Providing a closed-form solution suitable for a specific scenario about the partial compression offloading but not for general scenarios; reducing the end-to-end latency.
Energy cosumption	Energy efficient Multi-resource computation Offloading strategy task scheduling algorithm [30]	Yes	Medium	ES/CC	Comprehensively considering the workload conditions among mobile devices, edge servers and cloud centers; Having stable convergence speed and reducing the power consumption effectively in a specific scenario.
	Ordinal Optimization-based Markov Decision Process [43]	Unc	High	ES/CC	Being effective and efficient; Making good tradeoff between delay time and energy consumption.
	Ben’s genetic algorithm [56]	N-O	Medium	ES	Effectively solving the problem of choosing which edge server to offload, and minimizing the total energy consumption, but working only for a simple M/M/1 queue model
	Algorithms for partial and binary offloading with energy consumption optimization [57]	Yes	High	ES	Joint computation and communication cooperation by considering both partial and binary offloading cases Reducing the power consumption effectively; obtaining the optimal solution in a partial offloading case
	Artificial fish swarm algorithm [58]	Yes	Medium	ES	Guaranteeing the global optimization, strong robustness and fast convergence for a specific problem and reducing the power consumption
Delay/energy consumption	Multidimensional numerical method [17]	Yes	High	ES	Establishing the conditions under which total or no offloading is optimal; Reducing the execution delay of applications and minimizing the total consumed energy but failing to consider latency constraints
	Software defined task offloading/Task Placement Algorithm [59]	Yes	Medium	ES	Solving the computing resource allocation and task placement problems; Reducing task duration and energy cost compared to random and uniform computation offloading schemes by considering computation amount and data size of a task in a software defined ultra network.
	Energy-aware mobility management algorithm [60]	N-O	High	ES	Making good tradeoff between delay time and energy consumption Dealing with various practical deployment scenarios including BSs dynamically switching on and off, but failing to consider the capability of a cloud server.
	Lyapunov Optimization on Time and Energy Cost [61]	Yes	High	ES/CC	Taking full advantages of green energy without significantly increasing the response time and having better optimization ability

## References

[1] Patidar S., Rane D., Jain P. A Survey Paper on Cloud Computing. Proceedings of the 2012 Second International Conference on Advanced Computing & Communication Technologies, Institute of Electrical and Electronics Engineers (IEEE).

[2] Moghaddam F.F., Ahmadi M., Sarvari S., Eslami M., Golkar A. Cloud computing challenges and opportunities: A survey. Proceedings of the 2015 1st International Conference on Telematics and Future Generation Networks (TAFGEN), Institute of Electrical and Electronics Engineers (IEEE).

[3] Varghese B., Wang N., Barbhuiya S., Kilpatrick P., Nikolopoulos D.S. Challenges and Opportunities in Edge Computing. Proceedings of the 2016 IEEE International Conference on Smart Cloud (SmartCloud), Institute of Electrical and Electronics Engineers (IEEE).

[4] Shi W., Cao J., Zhang Q., Li Y., Xu L. (2016). Edge Computing: Vision and Challenges. IEEE Internet Things J..

[5] Rincon J.A., Guerra-Ojeda S., Carrascosa C., Julian V. (2020). An IoT and Fog Computing-Based Monitoring System for Cardiovascular Patients with Automatic ECG Classification Using Deep Neural Networks. Sensors.

[6] Liu B. (2019). Research on collaborative scheduling technology Based on edge computing. Master’s Thesis.

[7] Jiao J. (2018). Cooperative Task Scheduling in Mobile Edge Computing System. Master’s Thesis.

[8] Zhao J., Li Q., Gong Y., Zhang K. (2019). Computation Offloading and Resource Allocation for Cloud Assisted Mobile Edge Computing in Vehicular Networks. IEEE Trans. Veh. Technol..

[9] Lyu X., Ni W., Tian H., Liu R.P., Wang X., Giannakis G.B., Paulraj A. (2017). Optimal Schedule of Mobile Edge Computing for Internet of Things Using Partial Information. IEEE J. Sel. Areas Commun..

[10] Mao Y., Zhang J., Letaief K.B. Joint Task Offloading Scheduling and Transmit Power Allocation for Mobile-Edge Computing Systems. Proceedings of the 2017 IEEE Wireless Communications and Networking Conference (WCNC), Institute of Electrical and Electronics Engineers (IEEE).

[11] Tao X., Ota K., Dong M., Qi H., Li K. (2017). Performance Guaranteed Computation Offloading for Mobile-Edge Cloud Computing. IEEE Wirel. Commun. Lett..

[12] Kim Y., Song C., Han H., Jung H., Kang S. (2020). Collaborative Task Scheduling for IoT-Assisted Edge Computing. IEEE Access.

[13] Wang S., Zafer M., Leung K.K. (2017). Online placement of multi-component applications in edge computing environments. IEEE Access.

[14] Zhao T., Zhou S., Guo X., Zhao Y., Niu Z. A Cooperative Scheduling Scheme of Local Cloud and Internet Cloud for Delay-Aware Mobile Cloud Computing. Proceedings of the 2015 IEEE Globecom Workshops (GC Wkshps), Institute of Electrical and Electronics Engineers (IEEE).

[15] Kao Y.-H., Krishnamachari B., Ra M.-R., Bai F. Hermes: Latency optimal task assignment for resource-constrained mobile computing. Proceedings of the 2015 IEEE Conference on Computer Communications (INFOCOM), Institute of Electrical and Electronics Engineers (IEEE).

[16] Cuervo E., Balasubramanian A., Cho D., Wolman A., Saroiu S., Chandra R., Bahl P. Maui: Making smartphones last longer with code offload. Proceedings of the MobiSys, ACM.

[17] Munoz O., Pascual-Iserte A., Vidal J. (2015). Optimization of Radio and Computational Resources for Energy Efficiency in Latency-Constrained Application Offloading. IEEE Trans. Veh. Technol..

[18] Yang L., Cao J., Tang S., Han D., Suri N. (2014). Run Time Application Repartitioning in Dynamic Mobile Cloud Environments. IEEE Trans. Cloud Comput..

[19] Yang L., Cao J., Cheng H., Ji Y. (2015). Multi-User Computation Partitioning for Latency Sensitive Mobile Cloud Applications. IEEE Trans. Comput..

[20] Liu L., Chang Z., Guo X., Ristaniemi T. Multi-objective optimization for computation offloading in mobile-edge computing. Proceedings of the 2017 IEEE Symposium on Computers and Communications (ISCC).

[21] Carson K., Thomason J., Wolski R., Krintz C., Mock M. Mandrake: Implementing Durability for Edge Clouds. Proceedings of the 2019 IEEE International Conference on Edge Computing (EDGE), Institute of Electrical and Electronics Engineers (IEEE).

[22] Liu J., Mao Y., Zhang J., Letaief K.B. Delay-optimal computation task scheduling for mobile-edge computing systems. Proceedings of the 2016 IEEE International Symposium on Information Theory (ISIT), Institute of Electrical and Electronics Engineers (IEEE).

[23] Lazar A. (1983). The throughput time delay function of anM/M/1queue (Corresp.). IEEE Trans. Inf. Theory.

[24] Zhang G., Zhang W., Cao Y., Li D., Wang L. (2018). Energy-Delay Tradeoff for Dynamic Offloading in Mobile-Edge Com-puting System with Energy Harvesting Devices. IEEE Trans. Industr. Inform..

[25] Chen X. (2015). Decentralized Computation Offloading Game for Mobile Cloud Computing. IEEE Trans. Parallel Distrib. Syst..

[26] Chen X., Jiao L., Li W., Fu X. (2016). Efficient Multi-User Computation Offloading for Mobile-Edge Cloud Computing. IEEE/ACM Trans. Netw..

[27] Yuan H., Bi J., Zhou M., Liu Q., Ammari A.C. (2020). Biobjective Task Scheduling for Distributed Green Data Centers. IEEE Trans. Autom. Sci. Eng..

[28] Guo X., Liu S., Zhou M., Tian G. (2018). Dual-Objective Program and Scatter Search for the Optimization of Disassembly Sequences Subject to Multiresource Constraints. IEEE Trans. Autom. Sci. Eng..

[29] Fu Y., Zhou M., Guo X., Qi L. (2020). Scheduling Dual-Objective Stochastic Hybrid Flow Shop with Deteriorating Jobs via Bi-Population Evolutionary Algorithm. IEEE Trans. Syst. Man Cybern. Syst..

[30] Sheng Z., Pfersich S., Eldridge A., Zhou J., Tian D., Leung V.C.M. (2019). Wireless acoustic sensor networks and edge computing for rapid acoustic monitoring. IEEE/CAA J. Autom. Sin..

[31] Yang G., Zhao X., Huang J. (2019). Overview of task scheduling algorithms in cloud computing. Appl. Electron. Tech. J..

[32] Zhang P., Zhou M., Wang X. (2020). An Intelligent Optimization Method for Optimal Virtual Machine Allocation in Cloud Data Centers. IEEE Trans. Autom. Sci. Eng..

[33] Yuan H., Bi J., Zhou M. (2018). Spatial Task Scheduling for Cost Minimization in Distributed Green Cloud Data Centers. IEEE Trans. Autom. Sci. Eng..

[34] Yuan H., Zhou M., Liu Q., Abusorrah A. (2020). Fine-Grained Resource Provisioning and Task Scheduling for Heterogeneous Applications in Distributed Green Clouds. IEEE/CAA J. Autom. Sin..

[35] Alfakih T., Hassan M.M., Gumaei A., Savaglio C., Fortino G. (2020). Task Offloading and Resource Allocation for Mobile Edge Computing by Deep Reinforcement Learning Based on SARSA. IEEE Access.

[36] Yuchong L., Jigang W., Yalan W., Long C. Task Scheduling in Mobile Edge Computing with Stochastic Requests and M/M/1 Servers. Proceedings of the 2019 IEEE 21st International Conference on High Performance Computing and Communications, IEEE 17th International Conference on Smart City, IEEE 5th International Conference on Data Science and Systems (HPCC/SmartCity/DSS), Institute of Electrical and Electronics Engineers (IEEE).

[37] Li X., Wan J., Dai H.-N., Imran M., Xia M., Celesti A. (2019). A Hybrid Computing Solution and Resource Scheduling Strategy for Edge Computing in Smart Manufacturing. IEEE Trans. Ind. Inform..

[38] Zhang Y., Xie M. A More Accurate Delay Model based Task Scheduling in Cellular Edge Computing Systems. Proceedings of the 2019 IEEE 5th International Conference on Computer and Communications (ICCC), Institute of Electrical and Electronics Engineers (IEEE).

[39] Zhang Y., Du P. (2019). Delay-Driven Computation Task Scheduling in Multi-Cell Cellular Edge Computing Systems. IEEE Access.

[40] Zhang W., Zhang Z., Zeadally S., Chao H.-C. (2019). Efficient Task Scheduling with Stochastic Delay Cost in Mobile Edge Computing. IEEE Commun. Lett..

[41] Yuan H., Zhou M. (2020). Profit-Maximized Collaborative Computation Offloading and Resource Allocation in Distributed Cloud and Edge Computing Systems. IEEE Trans. Autom. Sci. Eng..

[42] Xu J., Li X., Ding R., Liu X. (2019). Energy efficient multi-resource computation offloading strategy in mobile edge computing. CIMS.

[43] Ning Z., Huang J., Wang X., Rodrigues J.J.P.C., Guo L. (2019). Mobile Edge Computing-Enabled Internet of Vehicles: Toward Energy-Efficient Scheduling. IEEE Netw..

[44] Li S., Huang J. Energy Efficient Resource Management and Task Scheduling for IoT Services in Edge Computing Paradigm. Proceedings of the 2017 IEEE International Symposium on Parallel and Distributed Processing with Applications and 2017 IEEE International Conference on Ubiquitous Computing and Communications (ISPA/IUCC), Institute of Electrical and Electronics Engineers (IEEE).

[45] Yoo W., Yang W., Chung J. Energy Consumption Minimization of Smart Devices for Delay-Constrained Task Processing with Edge Computing. Proceedings of the 2020 IEEE International Conference on Consumer Electronics (ICCE).

[46] Zhang Q., Lin M., Yang L.T., Chen Z., Khan S.U., Li P. (2018). A Double Deep Q-Learning Model for Energy-Efficient Edge Scheduling. IEEE Trans. Serv. Comput..

[47] Yang Y., Ma Y., Xiang W., Gu X., Zhao H. (2018). Joint Optimization of Energy Consumption and Packet Scheduling for Mobile Edge Computing in Cyber-Physical Networks. IEEE Access.

[48] Bi J., Yuan H., Duanmu S., Zhou M.C., Abusorrah A. (2020). Energy-optimized Partial Computation Offloading in Mobile Edge Computing with Genetic Simulated-annealing-based Particle Swarm Optimization. IEEE Internet Things J..

[49] Yu H., Wang Q., Guo S. Energy-Efficient Task Offloading and Resource Scheduling for Mobile Edge Computing. Proceedings of the 2018 IEEE International Conference on Networking, Architecture and Storage (NAS), Institute of Electrical and Electronics Engineers (IEEE).

[50] Mao Y., Zhang J., Song S.H., Letaief K.B. (2017). Stochastic joint radio and computational resource management for multi-user mobile-edge computing systems. IEEE Trans. Wirel. Commun..

[51] Dinh T.Q., Tang J., La Q.D., Quek T.Q.S. (2017). Offloading in Mobile Edge Computing: Task Allocation and Computational Frequency Scaling. IEEE Trans. Commun..

[52] Sen T., Shen H. Machine Learning based Timeliness-Guaranteed and Energy-Efficient Task Assignment in Edge Computing Systems. Proceedings of the 2019 IEEE 3rd International Conference on Fog and Edge Computing (ICFEC); Institute of Electrical and Electronics Engineers (IEEE).

[53] Sajjad H.P., Danniswara K., Al-Shishtawy A., Vlassov V. SpanEdge: Towards Unifying Stream Processing over Central and Near-the-Edge Data Centers. Proceedings of the 2016 IEEE/ACM Symposium on Edge Computing (SEC); Institute of Electrical and Electronics Engineers (IEEE).

[54] Dong Z., Liu Y., Zhou H., Xiao X., Gu Y., Zhang L., Liu C. An energy-efficient offloading framework with predictable temporal correctness. Proceedings of the SEC ’17: IEEE/ACM Symposium on Edge Computing Roc.

[55] Ren J., Yu G., Cai Y., He Y. (2018). Latency Optimization for Resource Allocation in Mobile-Edge Computation Offloading. IEEE Trans. Wirel. Commun..

[56] Wang J., Yue Y., Wang R., Yu M., Yu J., Liu H., Ying X., Yu R. Energy-Efficient Admission of Delay-Sensitive Tasks for Multi-Mobile Edge Computing Servers. Proceedings of the 2019 IEEE 25th International Conference on Parallel and Distributed Systems (ICPADS), Institute of Electrical and Electronics Engineers (IEEE).

[57] Cao X., Wang F., Xu J., Zhang R., Cui S. (2019). Joint Computation and Communication Cooperation for Energy-Efficient Mobile Edge Computing. IEEE Internet Things J..

[58] Zhang H., Guo J., Yang L., Li X., Ji H. Computation offloading considering fronthaul and backhaul in small-cell networks integrated with MEC. Proceedings of the 2017 IEEE Conference on Computer Communications Workshops (INFOCOM WKSHPS); Institute of Electrical and Electronics Engineers (IEEE).

[59] Chen M., Hao Y. (2018). Task Offloading for Mobile Edge Computing in Software Defined Ultra-Dense Network. IEEE J. Sel. Areas Commun..

[60] Sun Y., Zhou S., Xu J. (2017). EMM: Energy-Aware Mobility Management for Mobile Edge Computing in Ultra Dense Networks. IEEE J. Sel. Areas Commun..

[61] Nan Y., Li W., Bao W., Delicato F.C., Pires P.F., Dou Y., Zomaya A.Y. (2017). Adaptive Energy-Aware Computation Offloading for Cloud of Things Systems. IEEE Access.

[62] Sahni Y., Cao J., Yang L., Ji Y. (2020). Multi-Hop Offloading of Multiple DAG Tasks in Collaborative Edge Computing. IEEE Internet Things J..

[63] Sahni Y., Cao J., Yang L., Ji Y. (2020). Multi-Hop Multi-Task Partial Computation Offloading in Collaborative Edge Computing. IEEE Trans. Parallel Distrib. Syst..

[64] Zhang P., Zhou M., Fortino G. (2018). Security and trust issues in Fog computing: A survey. Futur. Gener. Comput. Syst..

[65] Wang X., Ning Z., Zhou M., Hu X., Wang L., Zhang Y., Yu F.R., Hu B. (2018). Privacy-Preserving Content Dissemination for Vehicular Social Networks: Challenges and Solutions. IEEE Commun. Surv. Tutorials.

[66] Huang X., Ye D., Yu R., Shu L. (2020). Securing parked vehicle assisted fog computing with blockchain and optimal smart contract design. IEEE/CAA J. Autom. Sin..

[67] Zhang Y., Du L., Lewis F.L. (2020). Stochastic DoS attack allocation against collaborative estimation in sensor networks. IEEE/CAA J. Autom. Sin..

[68] Zhang P., Zhou M. (2020). Security and Trust in Blockchains: Architecture, Key Technologies, and Open Issues. IEEE Trans. Comput. Soc. Syst..

[69] Oevermann J., Weber P., Tretbar S.H. (2021). Encapsulation of Capacitive Micromachined Ultrasonic Transducers (CMUTs) for the Acoustic Communication between Medical Implants. Sensors.

[70] Deng S., Zhao H., Fang W., Yin J., Dustdar S., Zomaya A.Y. (2020). Edge Intelligence: The Confluence of Edge Computing and Artificial Intelligence. IEEE Internet Things J..

[71] Fortino G., Messina F., Rosaci D., Sarne G.M.L. (2020). ResIoT: An IoT social framework resilient to malicious activities. IEEE/CAA J. Autom. Sin..

[72] Wang F.-Y. (2020). Parallel Intelligence: Belief and Prescription for Edge Emergence and Cloud Convergence in CPSS. IEEE Trans. Comput. Soc. Syst..

